# Validating an updated protocol for the novel object recognition task in young pigs

**DOI:** 10.3389/fnbeh.2025.1480389

**Published:** 2025-03-31

**Authors:** Rebecca K. Golden, Ryan N. Dilger

**Affiliations:** ^1^Neuroscience Program, University of Illinois Urbana-Champaign, Champaign, IL, United States; ^2^Division of Nutritional Sciences, University of Illinois Urbana-Champaign, Champaign, IL, United States; ^3^Department of Animal Sciences, University of Illinois Urbana-Champaign, Champaign, IL, United States

**Keywords:** paradigm design, object recognition, novelty preference, young pigs, behavior

## Abstract

The objective of this study was to validate equipment and procedures involved in implementing the novel object recognition (NOR) paradigm with young pigs. Two experiments were run with the intent of determining improvements to the original, high-throughput NOR paradigm design. The focus of these experiments was the impact of confounding factors on the main cognitive outcome, recognition index (RI). Experiment 1 utilized 13 pigs that all performed the NOR task following the original paradigm with the addition of 2 extra testing days. Results from this experiment indicated that one test day is sufficient for producing RI values that differ (*p* < 0.05) from chance performance, which was set at 0.50 given the use of two objects. Results also indicated that pigs may habituate to the task itself after 1 day of testing as RI values were not different (*p* > 0.05) from that of chance on test days 2 or 3. Experiment 2 utilized 13 male and 16 female pigs to determine sex differences in paradigm outcomes in addition to introducing home-cage enrichment. Results indicated sex differences in investigative behaviors despite both sexes producing RI values different from that of chance. The impact of home-cage enrichment was less discernable, but evidence suggests a lack of influence. Overall, the modifications to the NOR paradigm described herein reduced variability in the primary outcome, RI, and thereby improved sensitivity of the behavioral assay compared with the original paradigm.

## 1 Introduction

The novel object recognition (NOR) paradigm has been utilized with many species, starting with humans and rodents before being translated to pigs (Ennaceur and Delacour, 1988; Fantz, 1964; Moustgaard et al., 2002; Piaget and Inhelder, 1969). Since then, researchers have performed many different iterations of the task, ultimately precluding direct comparisons between studies. The NOR paradigm is a non-invasive and relatively high-throughput and low-labor task used to assess cognitive ability via the primary outcome measure of recognition index (RI). One of the biggest draws of using the NOR paradigm with pigs is the lack of training required, as it relies on a pig’s natural tendency to investigate novelty in its environment (Wood-Gush and Vestergaard, 1991). Animal usage of the NOR paradigm has focused largely on determining cognitive differences in impairment models, such as Alzheimer’s disease (AD), ischemic stroke, and acquired hydrocephalus (Kaiser et al., 2021; McAllister et al., 2021; Søndergaard et al., 2012). However, recent usage of the paradigm has been used in studies targeting nutritional intervention during critical brain development periods (Golden et al., 2024; Joung et al., 2020; Sutkus et al., 2022).

Use of the NOR paradigm with the pig model, as with other species, has seen many different iterations utilized, causing discrepancies across all phases of the task. For example, some studies have performed the NOR paradigm in the same room in which the pigs are housed (Gifford et al., 2007; Kaiser et al., 2021), relying on things such as curtains to mitigate sound and visual stimuli, while others have utilized a separate room for the paradigm (Buddington et al., 2018; Kornum et al., 2007; Parois et al., 2021). Discrepancies are also seen with the testing arenas. While some studies have utilized wood shavings or straw flooring, which can retain odors, (Gifford et al., 2007; Moustgaard et al., 2002), others have utilized raised, slatted flooring to mitigate odors (Fil et al., 2021a; Fil et al., 2021b; Fleming et al., 2019a; Golden et al., 2024). Discrepancies also span to the way the animal is introduced to the testing area with some studies utilizing a holding pen adjacent to the testing arena (Kouwenberg, 2008; Martin et al., 2015; Schmitt et al., 2019), which does not require researcher intervention for introduction to the testing arena, and others requiring direct placement by the researcher (Joung et al., 2020; Sutkus et al., 2022).

Procedural discrepancies are also present in the habituation and sample phases of the NOR paradigm. While some studies habituated their animals to the testing environment for multiple days ahead of testing (Kouwenberg, 2008; Martin et al., 2015; Parois et al., 2021; Schmitt et al., 2019), others provided little to no habituation (≤1 exposure) beforehand (Buddington et al., 2018; Fang et al., 2020; Kaiser et al., 2021). Exposure to familiar objects has also been done in a variety of ways. For example, some studies have exposed the animals to the familiar object in the same arena in which the test is performed (Golden et al., 2024; Joung et al., 2020; Sutkus et al., 2022), while others have exposed the animal to the familiar object in the animal’s home-cage (Gifford, 2005; Gifford et al., 2007). Test phase procedures also vary greatly, largely due to a wide range of delay period lengths (Fil et al., 2021a; Gifford et al., 2007; Kornum et al., 2007; Kouwenberg, 2008; Martin et al., 2015; Schmitt et al., 2019).

While many studies have utilized both male and female pigs, many have not provided results broken down by sex, despite evidence that male and female pigs behave differently during the paradigm (Fang et al., 2020; Fleming and Dilger, 2017). Other researchers have performed separate experiments for male and female pigs, causing a lack of direct comparison between the sexes (Kouwenberg, 2008). With evidence that male and female pigs produce differing outcomes on the NOR paradigm when tested in the same experiment, studies that test the two in separate experiments or do not assess their data utilizing sex as a main effect produce results that may unknowingly be driven by one sex.

The research described herein aims to bridge gaps in the literature due to paradigm inconsistencies by validating a standardized testing protocol that accounts for confounding factors. The procedure described herein is modified from previously detailed testing practices for studies utilizing young pigs in high-throughput behavioral testing (Fleming and Dilger, 2017). Two experiments were conducted to test potential confounding factors of the original procedure. Experiment 1 aimed to elucidate whether pigs habituate to the task and/or novelty in the environment by running 3 consecutive test days. We hypothesized that pigs would express novelty preference each test day and continue to investigate the objects with equal enthusiasm. Experiment 2 aimed to determine sex differences in performance during the NOR paradigm. We hypothesized that both sexes would express novelty preferences and behave similarly in their investigative behaviors.

## 2 Materials and methods

Two independent experiments were performed to test the hypotheses of whether pigs habituate to the NOR paradigm and whether sex influences cognitive outcomes from the NOR paradigm. Aside from minor procedural differences described in Section 2.5, experimental design, data collection, data processing, and statistical analyses were the same for both studies. The original paradigm design was documented in detail by Fleming and Dilger (2017).

### 2.1 Animals

All research and animal husbandry were in compliance with the National Research Council Guide for the Care and Use of Laboratory Animals and approved by the University of Illinois Urbana-Champaign Institutional Animal Care and Use Committee. Pigs were obtained from a commercial swine farm on postnatal day (PND) 2 and transported to the University of Illinois Piglet Nutrition and Cognition Laboratory (PNCL). All pigs received the same intake care of a 3-mL subcutaneous and 3-mL oral dose of *Clostridium perfringens* antitoxin C and D (Colorado Serum Company, Denver, CO). Pigs were individually reared in custom, artificial rearing home-cages, which have been previously described (Fil et al., 2021b). The home-cages allow pigs to see, hear, and smell other pigs but prohibit direct touching. Twice-daily health checks were performed to ensure animal well-being. To facilitate acclimation to the housing environment, pigs had access to electrolytes (Swine BlueLite; TechMix, Stewart, MN or Bounce Back, MannaPro®, LLC, St. Louis, MO) for the first week at PNCL. At the same time and throughout the remainder of the study, all pigs had *ad libitum* access to commercial milk replacer (TestDiet, Purina Mills, St. Louis, MO) for 20 h a day.

### 2.2 Arena design

**Figure 1:**
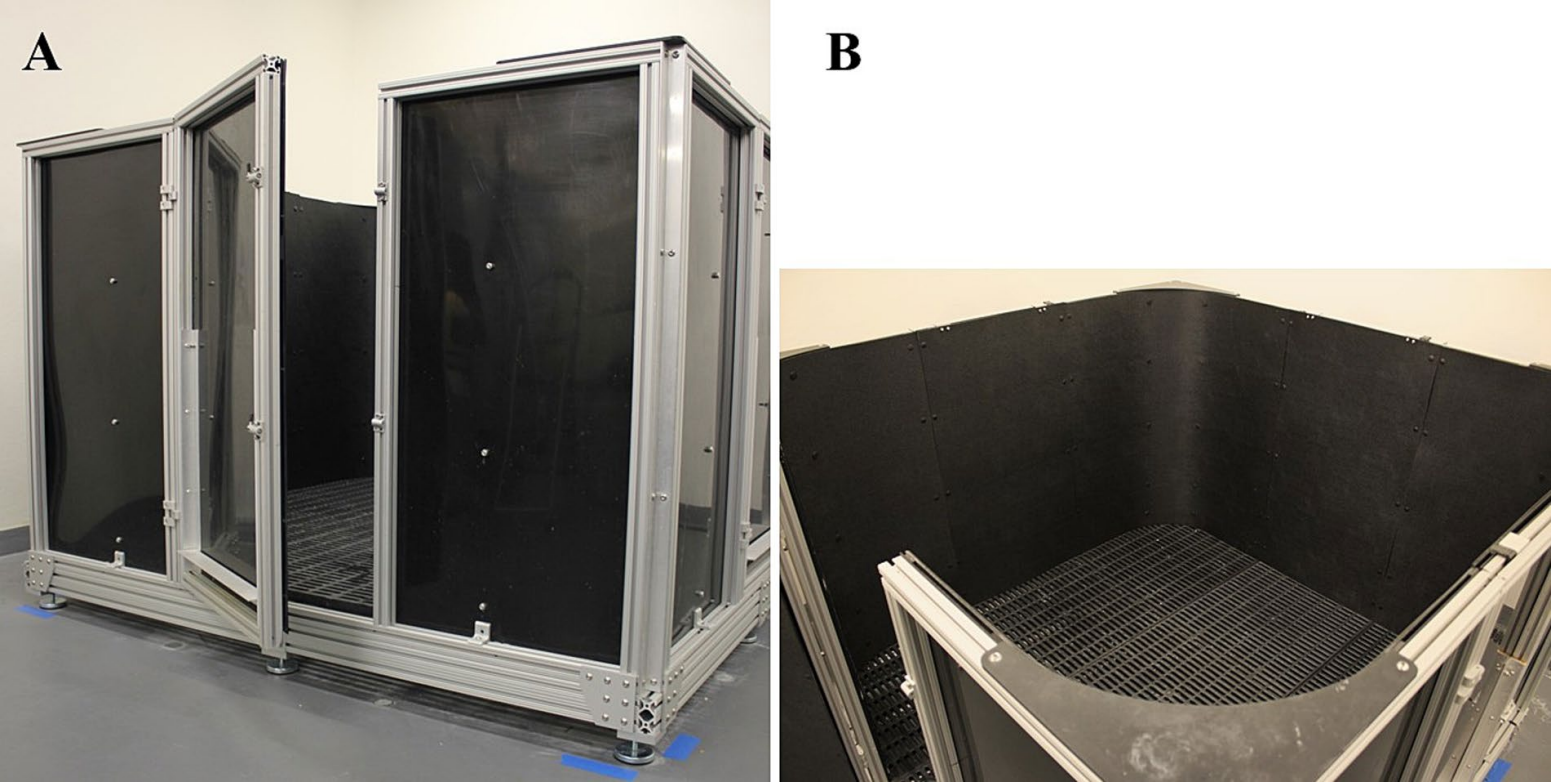
Novel object recognition paradigm testing arena for young pigs. **(A)** Front-facing view of the testing arena highlighting the outer structure as well as the hinged door entrance. Note the space between the floor and the bottom of the testing arena that allows for easy cleaning of equipment. **(B)** Perspective view of the arena highlighting the non-reflective surfaces of the walls and floors. Note the uniformity of the walls and the slatted flooring used to allow drainage.

**Figure 2:**
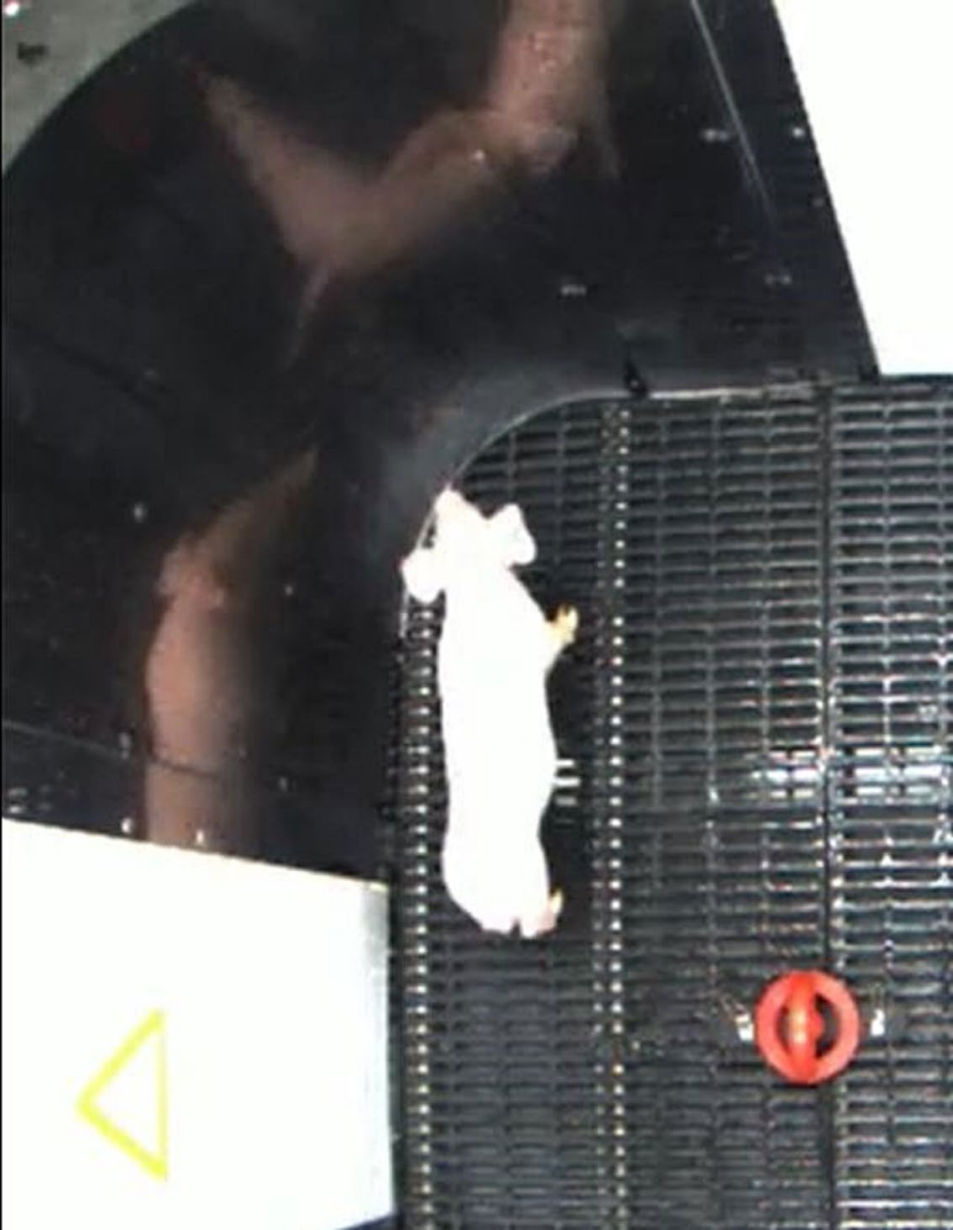
Reflections in a corner of a novel object recognition paradigm arena. Non-matte wall materials cause reflections that make it seem as though there are more pigs in the arena, which may take away attention from the intended objects.

The testing arena shown in Figure 1 was designed by ShapeMaster (Ogden, IL, United States). The base of the arena was made of raised, slatted flooring, 21 cm off the ground, for easy cleaning. The inner corners of the arena were radiused as internal evidence (not published) observed that pigs interact with squared corners in a more active manner, potentially confounding their investigation of the target objects. The inner area of the arena measured 177.8 cm × 177.8 cm × 116.8 cm (L × W × H). The inner walls were made of matte black styrene to reduce or eliminate light reflection. Reflective material can cause a situation where it appears as though there is more than one pig in the arena (Figure 2), which may also affect investigation of the target objects. The arena has 4 entry points, one on each side. Each entry point included both a hinged door (outward swing) and a guillotine-style door (vertical slide). The arena was in a separate room from the housing area to minimize external noise and elicit different behaviors than expected in the home cages. This arrangement also mitigated inadvertent exposure to the paradigm so pigs could not learn from seeing other pigs perform the task.

### 2.3 Camera and software

All paradigm phases (described below) were recorded by a single camera (Phoenix PHX064S-CC; LUCID Vision Labs, Richmond, BC, Canada) utilizing Motif recording software (Motif, version 5; Loopbio, GmbH, Vienna, Austria) for later analysis. The camera was centered above the arena 218.4 cm from the top of the arena floor and recorded at 30 fps. Videos were stored as MP4 files and uploaded to an online video annotation platform (Loopy, http://loopb.io/, Loopbio GmbH, Vienna, Austria) for analysis.

### 2.4 Habituation and sample phases

All pigs, regardless of experiment, experienced the same pre-test phase procedure beginning on PND 20, with the NOR paradigm conducted in three phases. First was the habituation phase, during which pigs were placed individually and directly into an empty arena by a researcher and allowed to explore for 10 min on each of two consecutive days. The pig was never exposed to the inside of the testing arena before this initial phase. Second was the sample phase, which was performed on the third day. The sample phase consisted of placing the pig back into the arena, which now contained two identical objects secured to the floor (denoted as ‘left-center’ and ‘right-center’), and allowing the pig to explore for 5 min. All pigs were exposed to the same object shape (described below) during the sample phase. The arena and objects were cleaned with diluted bleach and water between each pig each day to eliminate odor and excrement.

### 2.5 Test phase

**Figure 3:**
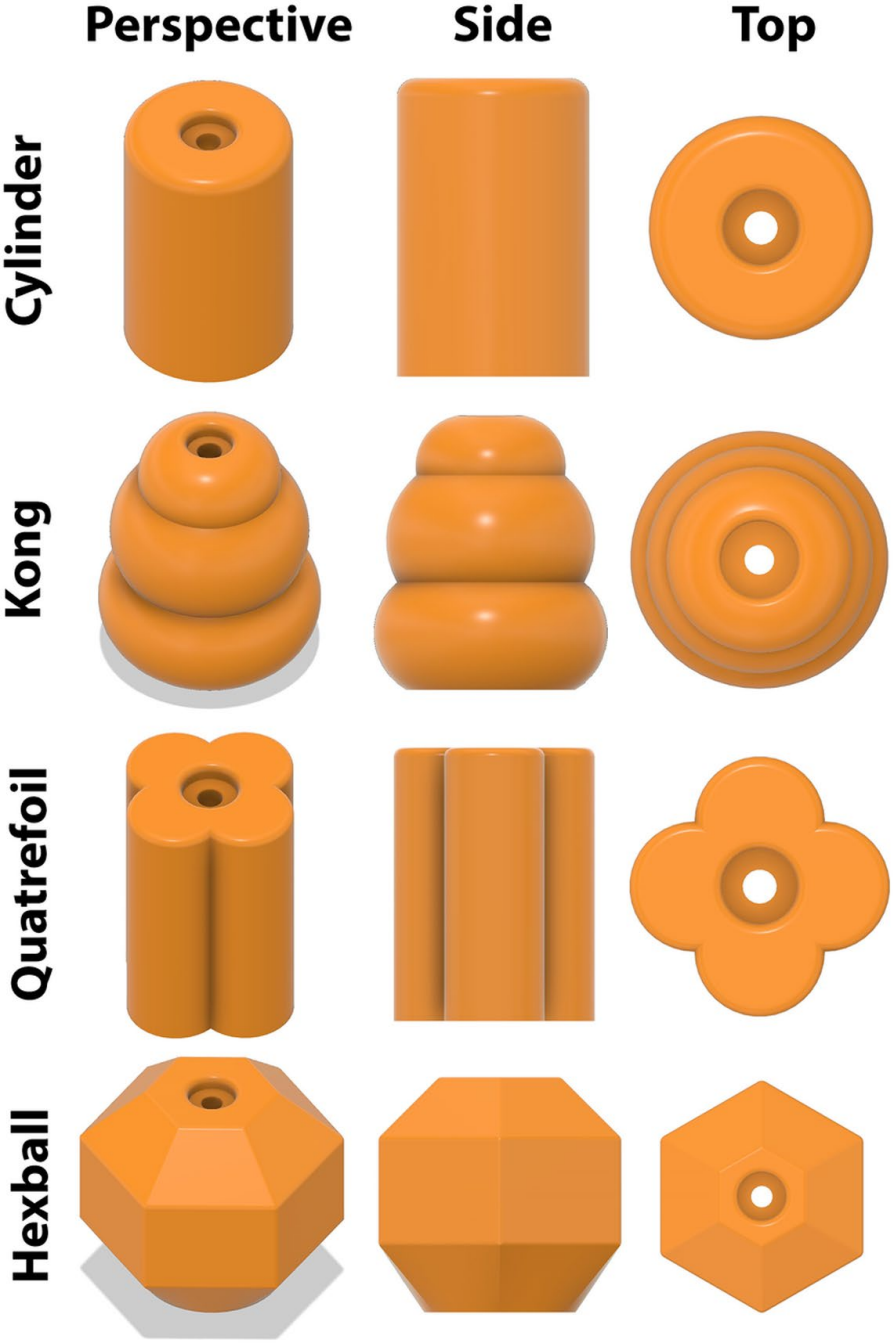
3D renderings of objects used for the novel object recognition paradigm. Four objects were designed to be different enough to be distinguishable but similar enough to not introduce confounding factors. Objects were composed of orange PETG filament (i.e., durable plastic) with a center hole for securing objects to a mounting plate attached to the arena floor. The top of the object was also recessed to prevent pigs from accessing the mounting bolt and thereby eliminate the ability to interact with anything but the object.

**Figure 4:**
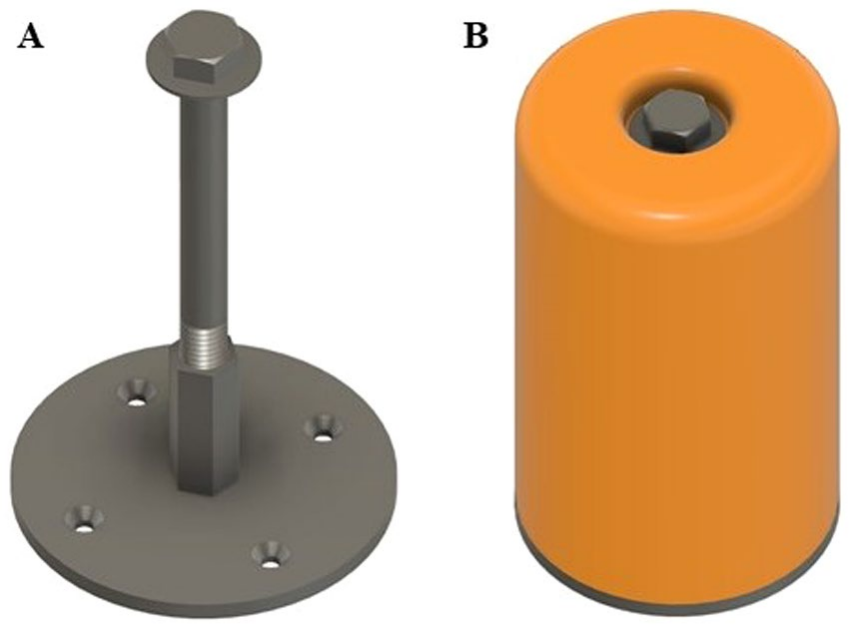
Securing system for objects used in the novel object recognition paradigm with young pigs. **(A)** The internal structure included a bolt the length of the object that secured into a hexagonal threaded nut welded to a base plate. The mounting plate was secured to the arena floor using bolts that threaded into brackets installed beneath the floor (i.e., nothing exposed above the flooring). **(B)** An object placed onto the base could be completely tightened to the point of becoming stationary. The object completely covers the base and hides the securing system, thereby mitigating any interactive potential.

Regardless of experiment, after a 48-h delay (i.e., paradigm day 5; PND 24), pigs began the third, or test, phase of the NOR paradigm. During a test day, pigs were placed into the arena, this time containing one object to which they were previously exposed during the sample phase (familiar object) plus one novel object. Three different novel objects were counterbalanced and randomly assigned to pigs for both studies. Objects were always the same color, composed of the same material (orange PETG filament; durable plastic), and were identical in height. Thus, objects only differed in shape (Figure 3). Stereolithography (STL) files can be found in the Supplemental materials for 3D printing applications. Shapes differed enough to be distinguishable, but not so much as to introduce biases due to “playability” (i.e., one object was not more “fun” to interact with). Previous research has suggested that object preference can supersede the intended goal of a paradigm, highlighting the importance of equally interesting objects (Kornum et al., 2007; Smith and Sheya, 2010; Van de Weerd et al., 2003). All objects were secured to the floor and completely immobile, with the pig not being able to access any component other than the object itself. As such, objects were mounted using a bolt that was accepted by a mounting plate containing a hexagonal threaded nut (Figure 4). Thus, objects remained securely fastened throughout testing and did not expose any mounting hardware to eliminate confounding factors that were previously identified (Golden and Dilger, 2024). Pigs were allowed to explore for 5 min and recorded throughout as described above.

#### 2.5.1 Experiment 1: Assessing NOR paradigm compliance

The aim of Experiment 1 was to assess performance on the NOR paradigm across multiple testing days. This was done with the intention of answering two main questions: (1) is one day of testing enough to elicit a full novelty investigation and (2) do pigs eventually lose interest in cooperating with the intent of the paradigm. As such, 13 male pigs performed the test phase of this experiment, which consisted of performing the test day described above on 3 consecutive days. The familiar object was the same across all 3 days, but the novel object was changed each day to mitigate familiarity biases. Similarly, the side of the arena on which the novel or familiar object was presented was counterbalanced to mitigate inherent location preferences. The arena and objects were, once again, cleaned with diluted bleach and water between each pig each day. All phases of the experiment were conducted at the same time of day and in the same pig order.

#### 2.5.2 Experiment 2: Influence of sex and home-cage enrichment on NOR paradigm outcomes

For this experiment, 13 intact male and 16 female pigs performed the test phase of this experiment. The test day procedure followed that as described above, where pigs were reintroduced to the arena now containing one familiar object and one novel object and allowed to explore for 5 min. The side on which the familiar and novel objects were presented was once again counterbalanced and the arena/objects cleaned between pigs. Unlike Experiment 1, however, pigs in Experiment 2 were only subjected to a single testing day. For this experiment, pigs also had access to home-cage enrichment prior to performing the NOR paradigm. The objects utilized as home-cage enrichment differed from the test objects in color, material, size, shape, texture, and mobility as to not introduce object familiarity. Unlike the paradigm objects, home-cage enrichment objects were purple, durable rubber dog chew toys in the shape of a football with cutouts in it. These objects were also attached to the floor by a plastic chain in order to allow movement. Enrichment objects were always present in home-cages beginning on PND 3 except for ~30 min each morning, during which time the objects were being cleaned.

### 2.6 Video processing

Videos for both experiments were analyzed by a single, trained, unbiased observer. Videos were recorded at 30 fps and the video annotation platform allowed for frame-by-frame analysis. Behavior scoring for these experiments was based on criteria used by Fleming and Dilger (2017). Investigations of the objects were identified based on position and movement of the pig’s snout (i.e., rooting behavior). An investigation event began when the pig’s snout was 7.6 cm away from and directed toward the object, and subsequent frames confirmed intent to investigate. An investigation event ended when the snout turned away from the object. A single investigative event was determined by a “begin” marker, when the above criteria were met, and the immediate next “end” marker, when the snout turned away. The elapsed time between these two markers determined the duration of the investigation event. Investigation events were scored for each object individually. Raw data was then exported as a comma-separated values (CSV) file. The raw data contained the start and stop frame of each event, the start and stop time (based on video time) of each event, as well as the assigned event title. The raw data were then run through a pipeline that processed the data into a user-friendly format that could be used to assess the outcomes described below.

### 2.7 Outcomes and statistical analyses

Recognition index (RI) was the main NOR outcome, as it is meant to act as an indicator of cognitive ability. RI was calculated as the amount of time investigating the novel object over the total amount of investigation time of both objects. Exploratory behaviors were also quantified, such as latency to investigations, number of investigations, and amount of time spent investigating each object. Each of the exploratory behaviors were quantified per pig for the objects individually, as well as both objects combined for total measures. Outcomes were then averaged either across day (Experiment 1) or sex (Experiment 2).

Statistical analyses were based on Golden and Dilger (2024). As such, pig inclusion criteria required at least 5 s of investigation over at least 3 investigations of the novel object for exploratory behavior and RI analyses. Any data from a pig not meeting these criteria were removed from analyses. Remaining data were then analyzed via a one-way analysis of variance (ANOVA) using the mixed procedure in SAS (RRID:SCR_008567; version 9.3; SAS Inst. Inc., Cary, NC, United States). Mean RI values per group (i.e., day or sex) were then compared to that of chance performance (i.e., 0.50) via a one-sample *t*-test. Exploratory behaviors were subsequently binned per minute of testing to assess pig focus on the task and habituation to novelty. Prior to binning, any pig that did not investigate either object in any capacity was removed from analyses. Differences in exploratory behaviors per minute were determined via a one-sample *t*-test. The level of significance was set at *p* < 0.05 for all analyses.

## 3 Results

### 3.1 Experiment 1

A total of 13 pigs completed all three test days. However, with the application of the data inclusion criteria for exploratory behavior and RI, data from 11, 10, and 12 pigs were used for analysis of performance on days 1, 2, and 3, respectively. A main effect of day (*p* = 0.03; *F*_19,19_ = 4.05) was observed for RI with pigs producing higher RI values on day 1 than on day 2, and day 3 exhibiting an intermediary value (Table 1). The RI value on day 1 also differed [*p* = 0.001; *t*(10) = 4.11] from the chance performance value (0.50). Although neither differed from that of chance, day 2 and 3 RI values were numerically less than and greater than 0.50, respectively. A main effect of day (*p* < 0.05) was also observed for familiar object investigation time and familiar object mean investigation time, as well as total investigation time. In all cases, day 1 produced lower values than those of days 2 and 3, while days 2 and 3 did not differ from each other.

**Table 1:** Experiment 1: Exploratory behavior of 4-week-old pigs during three test days of the novel object recognition (NOR) task; comparison of test days.

	Test Day			Pooled SEM^1^	
Behavioral measures	1	2	3		*p*-value^2^
Sample size	11	10	12	–	–
Recognition index^†^	0.69^b†^	0.42^a^	0.57^ab^	0.07	**0.034**
Exploration of the *novel* object					
Object investigation time, s	23.02	22.13	28.46	5.39	0.627
Number of object investigations	8.1	7.9	9.4	1.97	0.578
Mean object investigation time, s	2.79	2.94	3.21	0.60	0.851
Latency to first object investigation, s	26.54	18.49	19.57	9.02	0.726
Latency to last object investigation, s	225.30	230.92	293.72	19.00	0.832
Exploration of the *familiar* object					
Object investigation time, s	8.60^a^	35.93^b^	30.56^b^	7.84	**0.039**
Number of object investigations	5.7	7.5	7.2	1.23	0.290
Mean object investigation time, s	1.44^a^	4.52^b^	4.09^b^	0.80	**0.028**
Latency to first object investigation, s	35.83	28.12	22.13	10.85	0.635
Latency to last object investigation, s	226.42	234.28	225.44	19.78	0.945
Exploration of *both* objects					
Object investigation time, s	31.44	57.98	59.02	9.94	0.080
Number of object investigations	13.8	15.3	16.6	2.00	0.524
Mean object investigation time, s	2.11^a^	3.69^b^	3.57^b^	0.47	**0.031**
Latency to first object investigation, s	5.69	10.58	8.37	4.34	0.701
Latency to last object investigation, s	255.20	268.93	260.48	11.10	0.699

^†^Recognition index value differs (*p* < 0.05) from that of chance (0.50). ^ab^Means lacking a common superscript letter within a row differ (*p* < 0.05). ^1^SEM, standard error of mean. ^2^*P*-value for the overall 1-way ANOVA, which included a single fixed effect of study/treatment group. Bolded values indicate significant results.

**Figure 5:**
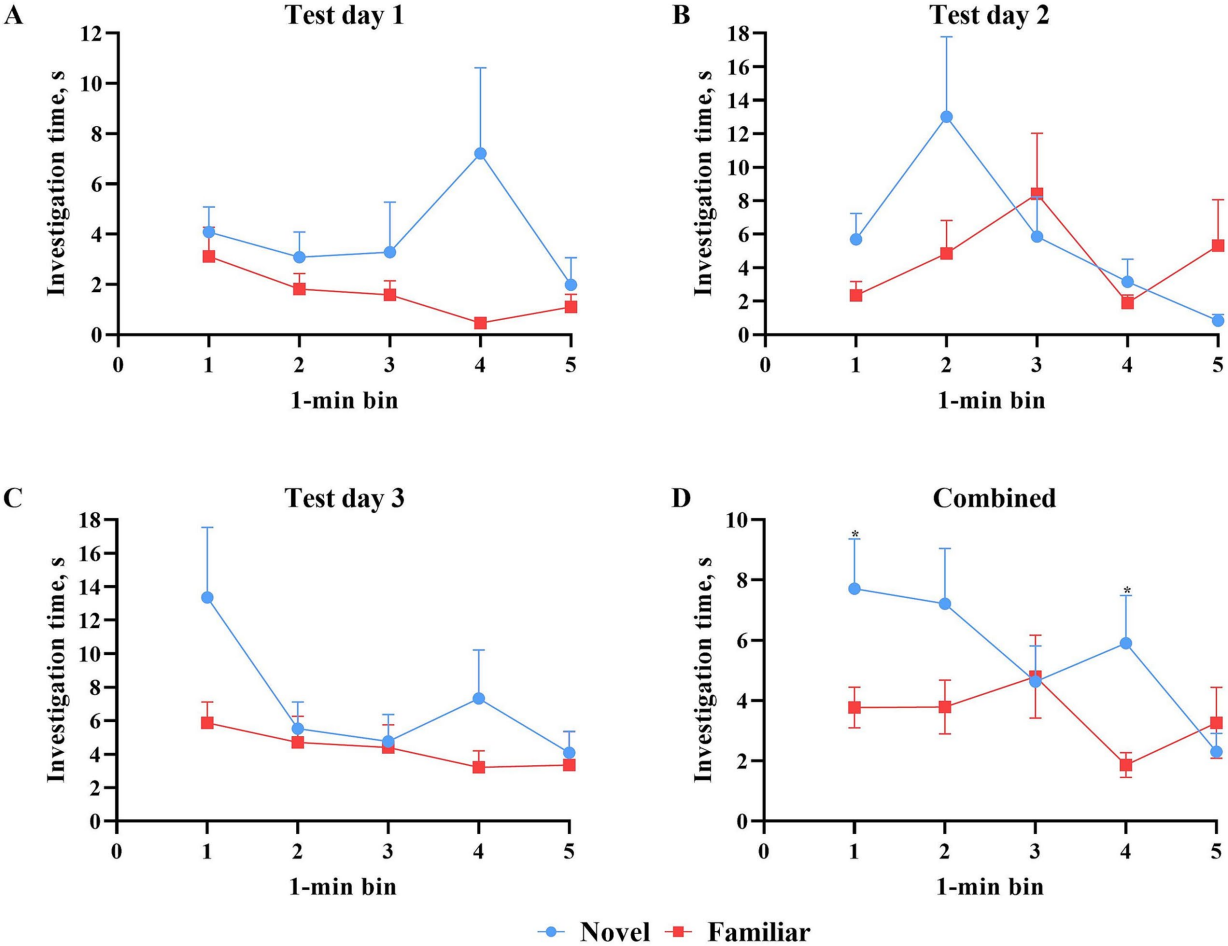
Investigation time (S) per 1-min bin of the novel and familiar objects during 3 test days of the novel object recognition paradigm. All error bars represent the standard error of the mean. **(A)** Novel versus familiar object investigation time (s) during the first test day. While the investigation time of the novel object was numerically greater, statistically, there was no difference in investigation time of the objects at any individual minute. **(B)** Novel versus familiar object investigation time (s) during the second test day. Which object was investigated more fluctuated from minute to minute, but at no single minute was the difference in investigation time significant. **(C)** Novel versus familiar object investigation time (s) during the third test day. While the investigation time of the novel object was numerically greater, statistically, there was no difference in investigation time of the objects at any individual minute. **(D)** Novel versus familiar object investigation time (s) averaged across all three test days. Which object was investigated more fluctuated from minute to minute. The difference in investigation time during minute four was significantly different with the novel object producing higher investigation time.

**Figure 6:**
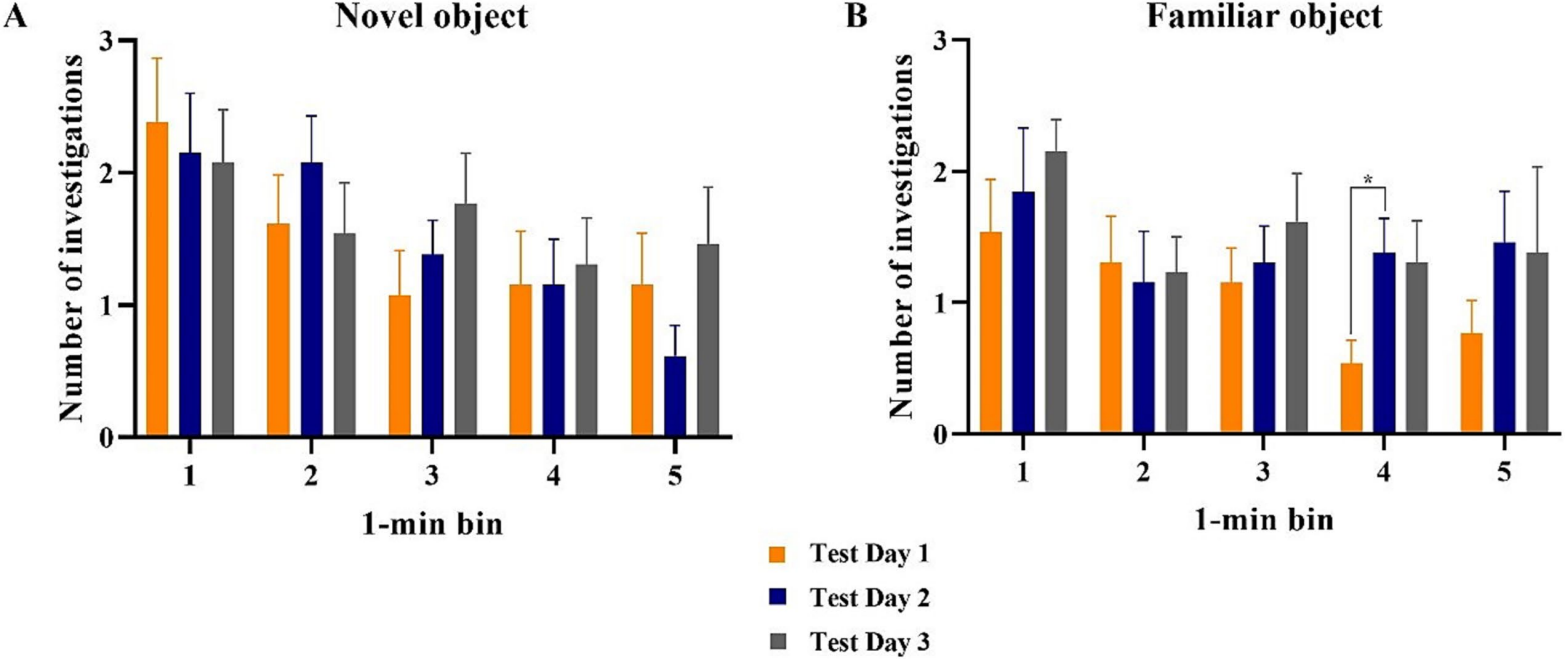
Number of investigations of the novel and familiar objects per 1-min bin during 3 test days of the novel object recognition paradigm. All error bars represent the standard error of the mean. **(A)** The novel object was investigated for the same amount of time during each minute across all 3 test days. **(B)** The familiar object was investigated for similar amounts of time across all 3 days for every minute except the 4th minute, during which pigs investigated the familiar object less on test day 1 than on test day 2 while test day 3 observed an intermediate amount of investigation time.

All data points were included in binning analyses, regardless of whether inclusion criteria were met. As such, each test day contained data from 13 pigs. Assessing test days independently, investigation time of the objects did not differ during any 1-min bin (Figure 5). However, mean investigation time (i.e., average of all 3 days) per minute revealed statistically more (*p* < 0.05) investigation of the novel object during minutes 1 and 4. Pigs investigated the novel object numerically more across the entire test on days 1 and 3, but not on day 2. There were no differences in the number of novel object investigations during any 1-min bin attributable to the effect of day (Figure 6). However, pigs did investigate the familiar object more times (*p* = 0.016) during minute 4 on test day 2 than on test day 1.

### 3.2 Experiment 2

A total of 13 intact male and 16 female pigs completed the sole test day for Experiment 2. However, after the application of the data inclusion criteria for exploratory behaviors and RI, data from 9 males and 11 females were used for data analysis. A main effect of sex was observed for RI with females producing higher [*p* = 0.03; *F*_18,18_ = 5.71; *t*(18) = 2.39] RI values than males (Table 2). That said, both males [*p* = 0.028; *t*(8) = 2.22] and females [*p* < 0.001; *t*(10) = 5.34] produced RI values that were greater than the chance performance value of 0.50. A sex difference was also observed in the number of familiar object investigations with males investigating more [*p* = 0.006; *F*_18,18_ = 9.59; *t*(18) = −3.10] than females. Similarly, a sex difference was observed for latency to the first investigation of the familiar object with males investigating quicker [*p* = 0.008; *F*_18,18_ = 8.83; *t*(18) = 2.97] than females.

**Table 2:** Experiment 2: Exploratory behavior of 4-week-old pigs during the test trial of the novel object recognition (NOR) task; comparison of sex.

	Sex		Pooled SEM^1^	
Behavioral measures	Male	Female		*p*-value^2^
Sample size	9	11	-	-
Recognition index	0.59^†^	0.75^†^	0.05	**0.028**
Exploration of the *novel* object				
Object investigation time, s	19.51	25.02	8.21	0.624
Number of object investigations	9.9	9.1	1.47	0.693
Mean object investigation time, s	2.21	2.31	0.53	0.894
Latency to first object investigation, s	11.13	29.80	11.13	0.238
Latency to last object investigation, s	253.17	246.85	17.47	0.791
Exploration of the *familiar* object				
Object investigation time, s	13.09	7.00	2.80	0.124
Number of object investigations	7.9	4.4	0.84	**0.006**
Mean object investigation time, s	1.80	1.48	0.41	0.564
Latency to first object investigation, s	10.94	114.45	25.84	**0.008**
Latency to last object investigation, s	229.21	248.42	18.92	0.461
Exploration of *both* objects				
Object investigation time, s	32.60	32.02	9.39	0.964
Number of object investigations	17.8	13.5	1.97	0.121
Mean object investigation time, s	2.03	2.05	0.45	0.977
Latency to first object investigation, s	4.30	25.93	10.47	0.144
Latency to last object investigation, s	259.31	273.98	14.77	0.471

^†^Recognition index value differs (*p* < 0.05) from that of chance (0.50). ^1^SEM, standard error of mean. ^2^*p*-value for the overall 1-way ANOVA, which included a single fixed effect of study/treatment group. Bolded values indicate significant results.

**Figure 7:**
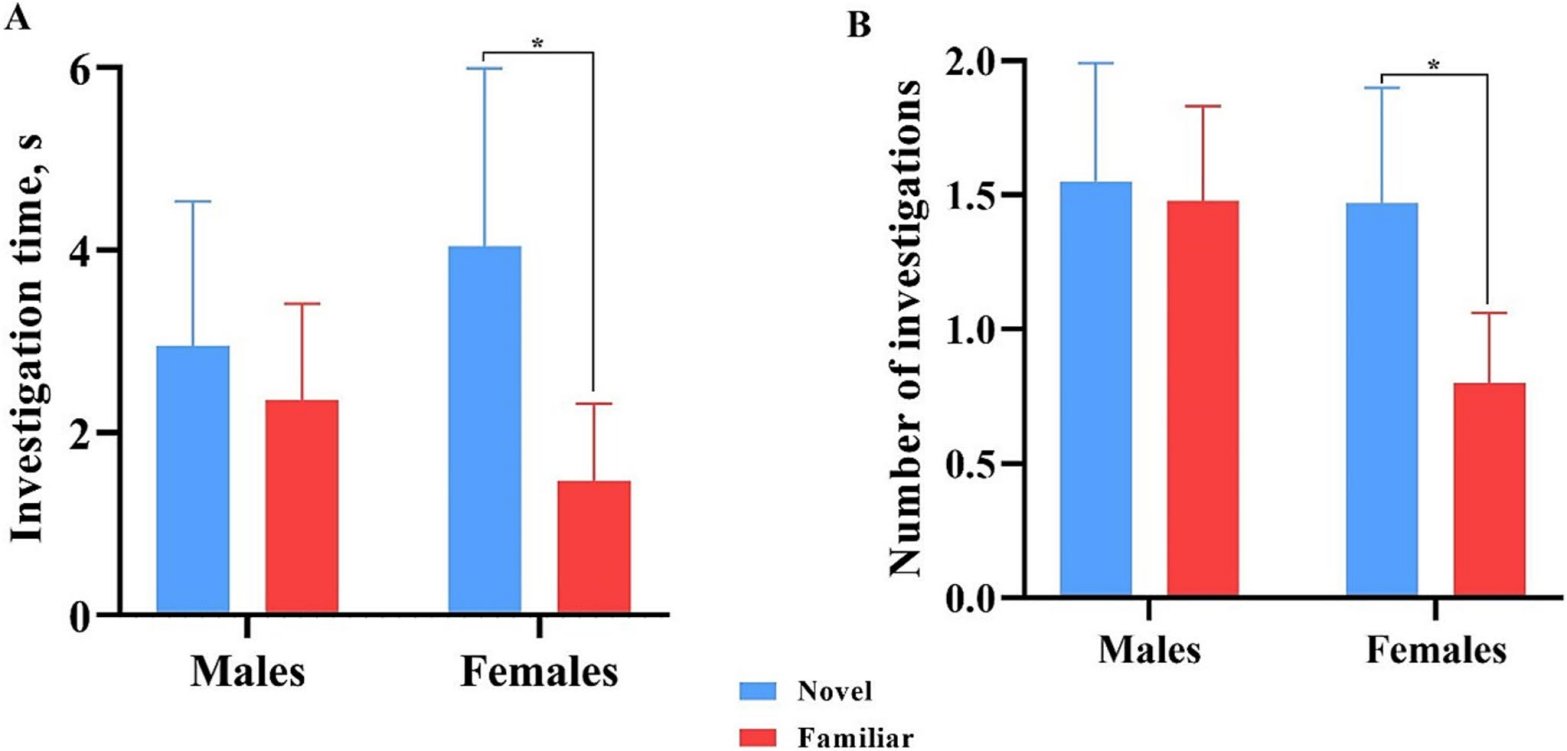
Investigative behaviors by male and female pigs toward the novel and familiar objects. All error bars represent the standard error of the mean. **(A)** Investigation time (s) by male and female pigs of the novel and familiar objects. Females spent significantly more time investigating the novel object than the familiar object. Males spent an equal amount of time investigating both. **(B)** Number of investigations of the novel and familiar objects by male and female pigs. Females produced significantly more investigations of the novel object than the familiar object. Males investigated both objects equally.

**Figure 8:**
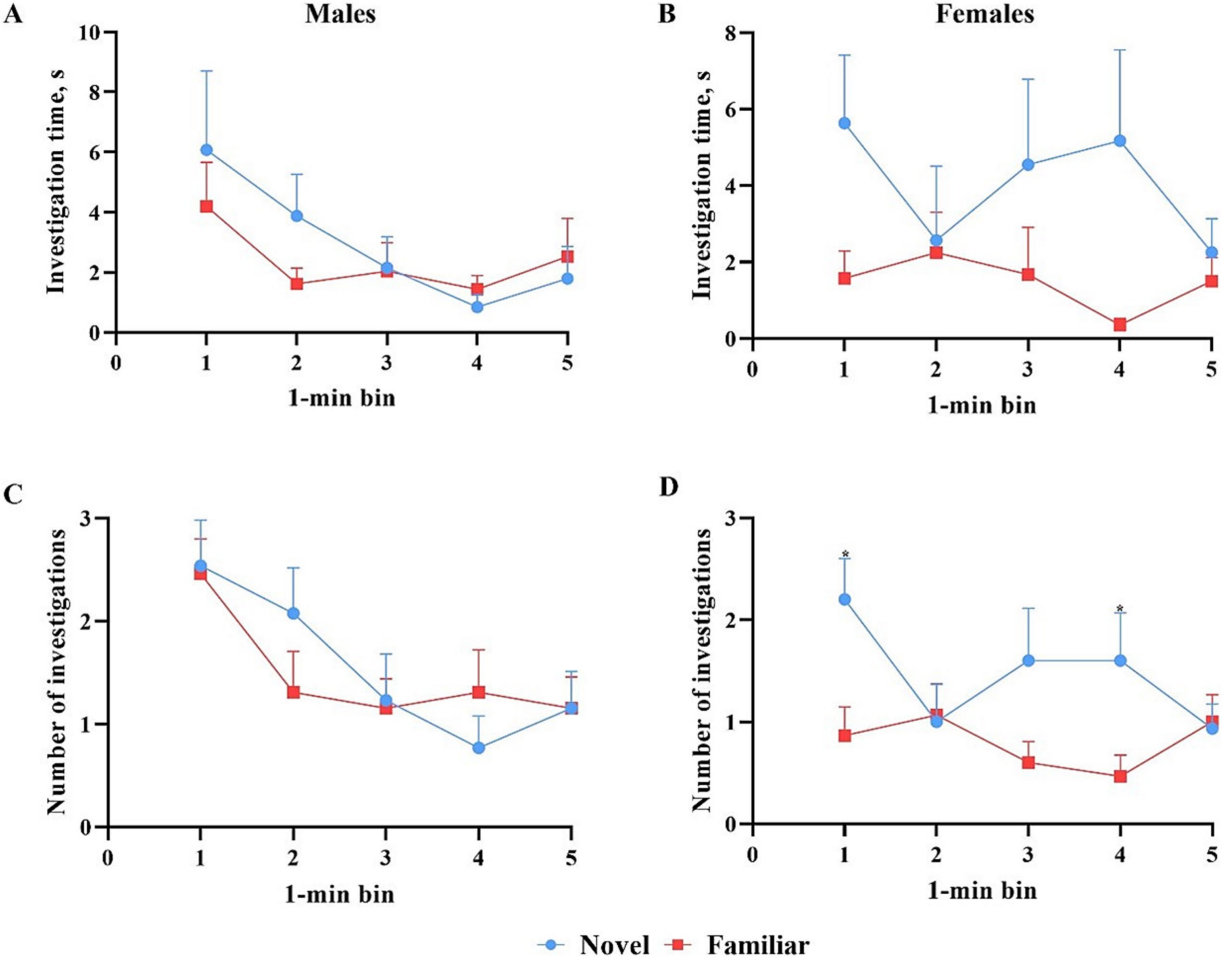
Investigative behaviors per 1-min bin by male and female pigs toward the novel and familiar objects. All error bars represent the standard error of the mean. **(A)** Investigation time (S) by male pigs of the novel and familiar objects. Male pigs investigated the novel object numerically more during the first 3 min but numerically less during the last two. **(B)** Investigation time (s) by female pigs of the novel and familiar objects. Female pigs investigated the novel object numerically more than the familiar object during each minute, while male pigs did not. **(C)** Number of investigations by male pigs of the novel and familiar objects. The number of investigations by male pigs of either object fluctuated from minute to minute with no significant differences. **(D)** Number of investigations by female pigs of the novel and familiar objects. Female pigs investigated the novel object significantly more during minutes 1 and 4 and numerically more during minute 3.

**Figure 9:**
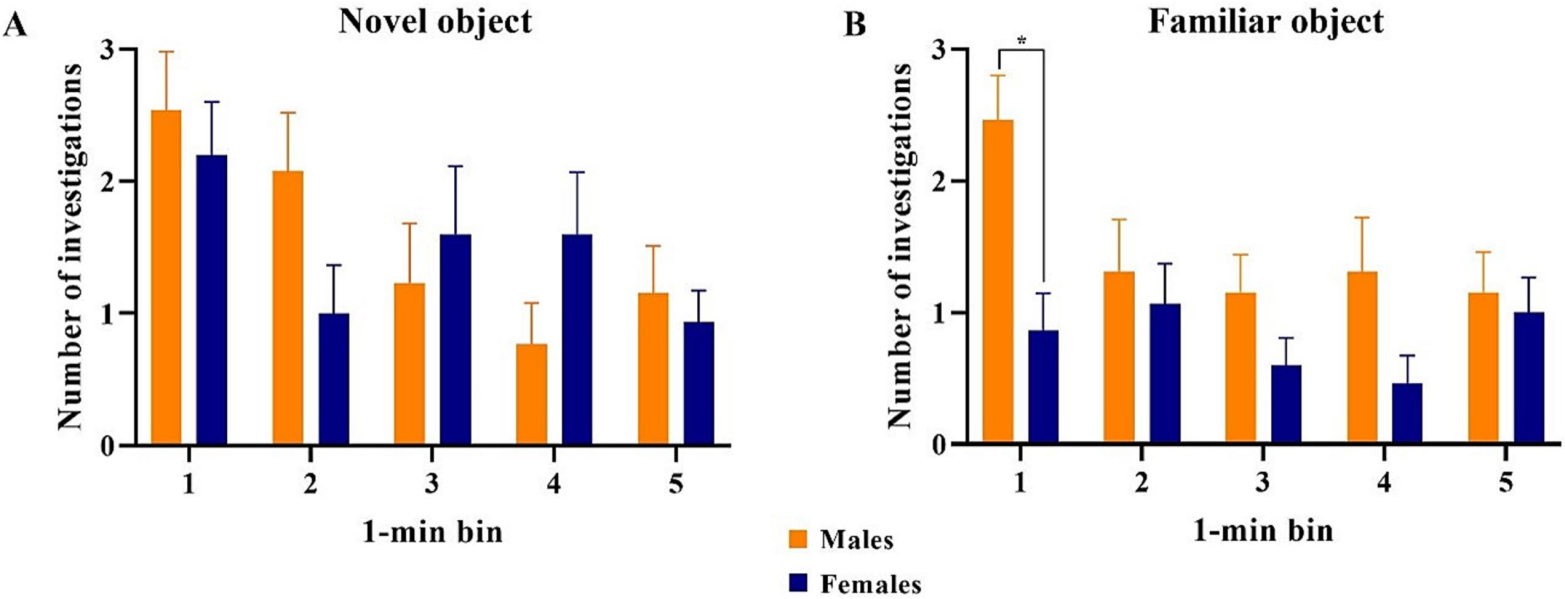
Comparison of the number of investigations by male and female pigs of the novel and familiar objects per 1-min bin. All error bars represent the standard error of the mean. **(A)** Male and female pigs expressed the same number of investigations of the novel object during each minute of the test. **(B)** Male pigs expressed a higher number of investigations of the familiar object than the female pigs during minute 1 of the trial but expressed equal numbers of investigation to the females for the remainder of the trial.

All data points were included in binning analyses, regardless of whether inclusion criteria were met, except for one female pig that did not investigate either object during the test phase. As such, 13 males and 15 females were included in the data analyses. Overall, females spent more time investigating (*p* = 0.009) and initiated more investigations (*p* = 0.004) with the novel object compared with the familiar object across the whole trial. Males investigated both objects equally (Figure 7). Males also did not investigate the objects differently (*p* > 0.05) in terms of time or number of investigations during any single minute (Figure 8). Numerically, females spent more time investigating the novel object compared with the familiar object for every minute of the test, but the number of investigations only differed by object during the 1st (*p* = 0.015) and 4th (*p* = 0.046) minutes of the trial. Males and females exhibited the same (*p* > 0.05) number of investigations of the novel object during any given minute of the test day (Figure 9). However, males investigated the familiar object more times (*p* = 0.002) during the 1^st^ minute than females.

## 4 Discussion

The work described herein is meant to improve upon previous research from our lab and establish best practices for performing the NOR paradigm with young pigs. In conjunction with determined statistical analyses (Golden and Dilger, 2024), this work details the hardware, software, and procedural steps for establishing a standardized method of running the NOR paradigm with young pigs to reduce interpretive confounds between independent studies. A paper by Fleming and Dilger (2017) established the foundational methods for high-throughput cognitive assessment of young pigs using the NOR paradigm by modifying the original translation of the NOR paradigm for pigs done by Moustgaard et al. (2002). Since then, that procedure has been utilized numerous times, but has not often resulted in identifying differences due to independent factors. As such, potential confounding factors and limitations were tested to determine their impact on cognitive and exploratory outcomes.

### 4.1 Experiment 1

In Experiment 1, habituation to novelty in the environment and to the paradigm itself was tested by performing the established test day procedure on three consecutive days, as opposed to just one. Overall, pigs spent numerically less time investigating objects on the first test day than on the subsequent test days. Interestingly, pigs spent approximately equal amounts of time investigating the novel object on all 3 days. Also of note is the increase in investigation of the familiar object on days 2 and 3, suggesting that the increase in overall investigation time is predominantly due to increased investigation of the familiar object. The investigation times in conjunction with the RI values suggest that pigs may have more interest in novelty at first exposure than on subsequent exposures. In other words, pigs may have habituated to novelty in this particular environment after 1 day of exposure. A study utilizing the same 10 pigs with 3 different delay intervals (10 min, 1 h, and 24 h) found decreased recognition indices as pigs progressed across the intervals (Kornum et al., 2007). It is unclear from the reported results whether this was due to decreased investigation of the novel object or increased investigation of the familiar object. However, authors from this experiment, as well as others, have observed increased investigation of the familiar object after initial investigation of the novel object (Kornum et al., 2007; Wood-Gush and Vestergaard, 1991). Such interpretations can be corroborated by results from our experiment and suggest that pigs may habituate to the goal of the task upon multiple exposures to objects.

Binning results from this experiment observed that across test day 1, pigs spent numerically more time investigating the novel object compared to the familiar object. However, this effect was not repeated on test day 2 or 3. This agrees with Kornum et al. (2007), where a preference for the novel object was only observed during the first exposure (10-min delay) to the paradigm. While Kornum et al. (2007) argued that this phenomenon was due to decreased memory for the familiar object as the delay increased, an alternative interpretation involves loss of interest in, or habituation to, the paradigm itself. The idea of decreased memory for the familiar object as the delay interval increases comes from Ennaceur and Delacour (1988), who found decreased discrimination indices produced by rodents that were tested after 24 h, compared with those tested at 1 min or 1 h. However, it should be noted that a different group of rodents was used for each delay interval, whereas with Kornum and colleagues, the same pigs were tested at each delay. As such, the binning data from the pig experiment is a more appropriate comparator to that of our study.

Results from our experiment indicate that repeated exposure to the NOR paradigm may lead to habituation to novelty in the environment or habituation to the paradigm itself. Previous research suggests that pigs return to investigation of the familiar object after sufficient investigation of the novel object. However, further research is warranted to determine whether stress from repeated testing may influence paradigm outcomes. The RI values from this experiment suggest that despite exposure to different novel objects each day, the novelty of the paradigm, or the novelty of the objects themselves, had been exhausted. Further testing is warranted to corroborate these results, as well as to test whether the differences between the objects used are great enough to elicit novelty preference, regardless of repeated paradigm exposure.

### 4.2 Experiment 2

The aim of Experiment 2 was to determine sex differences in the results of the NOR paradigm, as well as the influence of enrichment object exposure prior to paradigm testing. Results of Experiment 1 indicated that one test day is sufficient, and may even be preferable, to elicit novelty preference. As such, the pigs in this experiment only performed the test day once. In Experiment 2, female pigs exhibited a higher RI value, a lower number of familiar object investigations, and a longer latency to first familiar object investigation than males. Both sexes also produced RI values that were above that of chance performance. Previous work observed that female pigs produce numerically higher RI values than males, although not statistically different (Fleming and Dilger, 2017). These authors also observed RI values produced by both male and female pigs to be significantly greater than that of the chance performance value after a 48-h delay interval. While Fleming and Dilger (2017) did not find a significant difference between sexes for the number of investigations of the familiar object, the latency to the first familiar object visit was trending (*p* = 0.053), with females taking longer to initiate the first investigation.

Results from this experiment observed that females investigated the novel object more than the familiar object, both in terms of time and number of investigations. Sex differences in cognitive ability have been suggested to be the result of brain development differences (Fleming and Dilger, 2017; Jiang et al., 2020). Specifically, the female pig brain tends to develop faster than the male pig brain, especially the hippocampus, which has a growth spurt around PND 21 for females and PND 28 for males (Conrad et al., 2012). This difference in hippocampal development timing may be influencing the results of the present experiment, given that testing began on PND 20, as research has shown that memory-dependent tasks require hippocampal activation (Eichenbaum, 2004; Scoville and Milner, 1957). Previous research supplemented sows with docosahexaenoic acid (DHA) and tested the impact of supplementation on the cognition of their offspring via NOR (Fang et al., 2020). While the DHA-supplemented sow group of male offspring spent more time investigating the novel object, they did not produce a significantly different number of investigations of the objects. That said, results indicated that female offspring of DHA-supplemented sows did investigate the novel object significantly more than the familiar object, both in terms of time and number of investigations. Fang et al. (2020) performed behavior testing beginning on PND 18, before the hippocampal growth spurt for either sex. However, previous research has suggested that DHA supplementation can increase recognition memory (Buddington et al., 2018), which may have counteracted the effects of the underdeveloped hippocampus, but only in female pigs, who typically experience an earlier hippocampal growth spurt.

By binning results with respect to time, we observed that males investigated both objects similarly across the entire test day, while females investigated the novel object significantly more during specific minutes of the trial. The investigative behaviors expressed by male pigs are concurrent with a study by Gifford (2005) in which short exposure (10 min) to the familiar object in combination with a long delay period (5 days) produced null preference toward either object across the test trial. Although Gifford did utilize both male and female pigs, the results were not discriminated by sex, so it is impossible to determine which sex was driving these results, if either. Both male and female pigs in our experiment expressed peak investigation toward the novel object during the first minute of the trial. Previous research has found that pigs express peak investigative behaviors early in the trial before decreasing investigation for the remainder of the trial (Gifford et al., 2007; Wood-Gush and Vestergaard, 1991). Male pigs followed this pattern more strictly than female pigs, which initially investigated the novel object more, decreased investigation in the 2nd minute, increased investigation of the novel object again during the 3rd and 4th minutes, and finally decreased investigation again during the 5th minute. Previous research has suggested that 10 min may not be great enough exposure to the familiar object for pigs to gather a stable memory of the familiar object (Gifford et al., 2007). This may explain the pattern of behavior expressed by the male pigs, as they may not have had a strong enough memory of the familiar object to show preference toward the novel object during any individual minute. This may also explain the investigative behavior of the female pigs. It is possible that, in combination with known brain development patterns, the female hippocampus was developed enough to retain trace memory for the familiar object but still required reacquisition to the familiar object. In other words, it is possible that female pigs initially identified the novel object as novel in the 1st minute, but then needed to confirm their recognition of the familiar object in the 2nd minute. The investigative behaviors for the remainder of the test day follow a more expected pattern of peak investigation of the novel object before decreased investigation.

In addition to testing for sex differences in the NOR paradigm, Experiment 2 also introduced home-cage enrichment. Due to the limited number of pigs, a 2 × 2 (i.e., sex × home-cage enrichment) design could not be utilized for this experiment, and direct comparisons across experiments would be inappropriate. However, anecdotally, investigative behaviors from this experiment resembled those of test day 1 from Experiment 1. While male pigs in this experiment tended to initiate first interaction with an object (novel, familiar, or both) quicker and initiate last interaction with an object later in the trial than the male pigs from Experiment 1, the differences do not appear to be significant. Females in Experiment 2 tended to initiate the first and last interactions later than the pigs in Experiment 1 during test day 1, but again, the differences do not seem to be drastic. The minor latency discrepancies considered may not be related to novelty, but rather, may relate back to a theory posed by Hessing et al. (1993) in which authors assert that latency to novelty investigation may be related to proactive or reactive pig personality types rather than to novelty preference. In the case of Experiment 2, males may be considered more proactive, while females are considered more reactive. Thus, interpretation of our current findings aligns with previous research that focused on the direct impact of home-cage enrichment on pig behavior (Hill et al., 1998). While this study did not focus on novelty recognition, it did introduce novelty into the pig’s environment by way of a stationary human. The design of the study included various control and treatment groups made of combinations including or devoid of daily human interaction and access to home-cage enrichment. Results of this study indicated that behaviors toward environmental novelty were not different between treatment groups. The authors surmised that these results may be an indicator of a pig’s ability to adapt to new environments. Given that the NOR paradigm performed in Experiment 2 was carried out in an environment distinctly different from that of the home-cages, it is possible that the “playful” behaviors learned with the home-cage enrichment were non-transferrable and the multiple exposures to the testing environment prior to introduction of objects caused an adaptation to the environment that lent itself to uncertainty toward environmental stimuli.

Results from this experiment indicate that male and female pigs expressed investigative behavioral differences when performing the NOR paradigm. However, both sexes expressed recognition of the familiar object and, therefore, preference for the novel object during the test day. This experiment was conducted around the time of the female pig hippocampal growth spurt, which occurs earlier than for males. Future studies are warranted to determine sex differences when both sexes have experienced similar brain development. Anecdotal evidence and previous research suggest that home-cage enrichment does not lead to behavioral responses toward novelty in the environment. However, future research is warranted to determine the direct impact of home-cage enrichment on NOR paradigm performance.

### 4.3 Comparison of results

The purpose of these experiments was to implement an updated testing paradigm and data processing pipeline in an effort to mitigate confounding factors found to be associated with original assay described by Fleming and Dilger (2017). Experiment 1 from our study mostly resembles that of Experiment 1 in the Fleming and Dilger paper, in which authors tested the same pigs in the NOR paradigm from PND 17–21 and again from PND 24–28. Of note, this iteration of the NOR paradigm utilized a 24-h delay, as opposed to the 48-h delay utilized in our current experiment. This version also applied different inclusion criteria than utilized in our experiment. That said, upon first exposure to the test phase (PND 21), pigs from this experiment produced a mean RI value of 0.65 with a coefficient of variation (CV) of 30%. Pigs on test day 1 (PND 24) from our experiment produced a mean RI value of 0.69 and a CV of 22%. These results would indicate that the application of the new inclusion criteria reduced RI variability from pigs’ first exposure to the NOR paradigm. However, upon repeated exposure to the paradigm (i.e., PND 28), pigs tested by Fleming and Dilger produced a mean RI value of 0.72 with a CV of 20%, whereas pigs in our study produced a mean RI value of 0.42 with a CV of 53% on test day 2 (PND 25) and a mean RI value of 0.57 with a CV of 43% on test day 3 (PND 26). While it may seem that these results are contradictory, discrepancies in the experimental design may be influencing the results. In our experiment, pigs only performed the habituation and sample phases once, and were exposed to 3 consecutive test days utilizing the same familiar object each time. In the case of Fleming and Dilger, pigs performed all three phases of the paradigm two times (one test day each time), with different object sets used for each iteration, meaning that the familiar and novel objects were different at both time-points. As such, it may be that either repeated exposure to the same familiar object or rapid repeated exposure to the test phase itself exhausted the focus of the pigs to perform the task and that a break and/or a different set of objects is required for repetition of the task.

Experiment 2 from our study is most comparable to Experiment 2 from Fleming and Dilger (2017) in which the authors tested for sex differences in outcomes from the NOR paradigm. Pigs in this experiment performed the test day on PND 21 whereas pigs in our study performed the test day on PND 24. That said, male pigs from the Fleming and Dilger experiment produced a mean RI value of 0.65 with a CV of 19% while female pigs produced a mean RI value of 0.63 and a CV of 27%. Male pigs from our experiment produced a mean RI value of 0.59 and a CV of 20% while female pigs produced a mean RI value of 0.75 and a CV of 20%. Results from this comparison indicate that the inclusion criteria used in our experiment are more effective at controlling RI variability for female pigs compared with inclusion criteria used in the original paradigm. These results may also indicate that, in general, data produced by male pigs is not as variable, given that the CV from both experiments are relatively low and similar despite the application of differing inclusion criteria.

As another comparator, a study by Fleming et al. (2019b) utilized the NOR paradigm to test for cognitive differences between control (i.e., non-supplemented) pigs and pigs supplemented with polydextrose and galactooligosaccharide (PDX/GOS). Pigs performed the test phase of the NOR paradigm on PND 28 or 29. Those that were tested on PND 29 were exposed to a similar cognitive task on PND 28, which may have impacted performance on the NOR paradigm on PND 29. That said, control pigs produced a mean RI value of 0.56 with a CV of 35% while PDX/GOS pigs produced a mean RI value of 0.73 with a CV of 13%. This study strictly utilized male pigs. As such, comparison of the control pigs’ results is most reasonable to the male pigs in Experiment 2 of our study, which produced a higher mean RI value with a lower CV. Results from our Experiment 1 indicated that repeated exposure may be detrimental to the intent of the NOR task and that data is most reliable from the first exposure. As such, when comparing the results of the first test day of our experiment to the control pigs in this study, the pigs from our study produced a higher mean RI value with a lower CV. The differences observed between the results from test day 1 of our experiment and the control pigs in this experiment may be linked to the inclusion criteria differing, indicating that the inclusion criteria used in our study are better at controlling RI variability. However, when considering the PND of testing, pigs from test day 3 of our study are closer in age to those in Fleming et al. (2019b). In this instance, pigs from our study produced a similar mean RI value with a higher CV. Both the Fleming and colleagues’ study and our own performed the test phase before the hippocampal growth spurt for male pigs, which is around PND 28. Given that dietary supplementation of PDX/GOS has been observed to promote brain and cognitive development (Waworuntu et al., 2014), this may be an explanation for the higher RI value with a lower CV produced by the treatment group compared with the control group and results from all three test days in our study.

A study by Golden et al. (2024) utilized the NOR paradigm to test for cognitive differences between control (i.e., non-supplemented) and sialyllactose (SL) supplemented uncastrated boars. Pigs in this study performed the test phase on PND 28. Control pigs produced a mean RI value of 0.63 with a CV of 39% while the SL pigs produced a mean RI value of 0.49 with a CV of 51%. Of note, this study was conducted over the course of a year and a half and utilized 5 individual cohorts, which may have introduced uncontrollable variability in the data. The same behavior researcher oversaw the running of the NOR paradigm in this study and both experiments in our paper. Comparing these results to the test day 1 results (RI = 0.69; CV = 22%) from Experiment 1 and the male pigs (RI = 0.59; CV = 20%) from Experiment 2, the results would indicate that consistent research personnel oversite does not reduce performance variability. Instead, results indicate that amendments to the hardware utilized improved (i.e., lowered) performance variability.

Overall, data from the first exposure of pigs to the NOR paradigm in our experiments (i.e., test day 1 from Experiment 1 and male and female pigs from Experiment 2) produced a more narrow range in variability (CV = 20–22%) than the original design (CV = 19–30%). These results indicate that improvements have been made to the original design and that the NOR paradigm may now be more sensitive to treatment differences.

## 5 Conclusion

Herein, we described in detail the equipment and procedures for conducting an improved version of the NOR paradigm for young pigs. Confounding factors from the original high-throughput paradigm design were identified and amended. Experiments similar to the original assay were then run to elucidate the effects of the modifications. Results from Experiment 1 indicated that multiple exposures to the paradigm may lead to a loss of focus in the paradigm and that one test day is sufficient for producing object recognition that differs from that of chance performance. Results from this experiment also produced RI data that was equal to or less variable than the original design. Experiment 2 provided evidence that sex differences in investigative behaviors existed, although both sexes produced RI values above that of chance performance. Similar to Experiment 1, this experiment resulted in equal or lower RI data variability. Internal evidence and prior research suggest that home-cage enrichment does not impact results from the NOR paradigm. Further research is warranted to directly test the impact of home-cage enrichment on investigative behaviors observed in the NOR paradigm. Overall, improvements to the original design of the NOR paradigm have been made, resulting in reduced data variability, and the methods described should be utilized for future studies.

## Data Availability

The raw data supporting the conclusions of this article will be made available by the authors, without undue reservation.
